# Discussion by the Harveian Society

**Published:** 1921-02-05

**Authors:** 


### THE FUTURE OF POOR-LAW INFIRMARIES.

### Discussion by the Harveian Society.

As a preliminary to the Harveian Society's adjourned discussion on the future of Poor-law infirmaries which took place at Paddington Town Hall on January 27, a letter was read from Colonel F. E. Fremantle, M.P., in which he claimed that the eventual aim to be urged in the discussion should be the provision in every unit of administration of a complete system for treatment and cure of the whole population, with as much freedom for patients and doctors and for nurses, chemists, and other agents as possible, also with as much payment to be made directly or indirectly by the patient as possible. But the State should be responsible for filling all the gaps and co-ordinating all the factors, institutions, and departments into an efficient and economical whole.

Dr. A. G. Stewart, Medical Superintendent of Paddington Infirmary, opened the discussion, and proceeded to defend the infirmaries from current misconceptions. He considered it untrue that the Poor-law infirmaries deal only with chronic cases or with the aged, or that the patients are mostly paupers, and an exaggeration to say that there is a prejudice against Poor-law institutions. In a recent year half the total cases admitted at Paddington have made a stay of less than one month, and three-quarters, a stay of less than two months. In that same year only 20 per cent. were over sixty years of age, and only 20 per cent. also were workhouse patients. The great majority of patients are hardworking folk whose need of relief is only due to their sickness, and 60 per cent. of them contribute towards their maintenance. While admitting that the equipment and staffing in Poor-law infirmaries has not always been ideal, Dr. Stewart resented the suggestion that they provide little more than food and shelter. Much highly-specialised medical and surgical work is done, and, in regard to the nursing, he maintained that Poor-law nurses in the separate infirmaries are as well trained, in private nursing, at any rate, as at the general hospitals.

He explained that both the general hospital and the infirmary have distinct futures. The former must consider more particularly the scientific research and teaching points of view, while the latter must tackle the economic problem, approaching its cases from the point of view, not of the specialist, but of the general practitioner. If these outlooks were realised, and especially if there were some central authority to co-ordinate the efforts on both sides, great improvements might be effected and money saved. He suggested that sundry part-time general practitioners should be on infirmary staffs, but only provided they demanded sufficient remuneration to justify them in giving the necessary time to the work.

Dr. Reginald Dudfield (Medical Officer of Health for Paddington) said that he is unable to appreciate the distinction between State or municipal assistance and Poor-law relief. Why should not the State or the municipality be looked upon as a national charity organisation society? To meet certain objections, however, he believes that the infirmaries might be transferred from the Poor Law to the municipal authorities. He dissents from the view that *ad hoc* authorities should be set up, and he believes the competent body in London to be the County Council. Also, it might be well to have, at the remodelled infirmaries, local practitioners as visiting physicians and surgeons, serving as such by rota. Every ratepayer should have the right of admission to such hospitals if the admission be certified as necessary by the practitioner in charge of the case. Such right should not involve free treatment in every case. The principle of assessment, as now enforced by the County Council for the treatment of the uninsured tuberculous, might be applied. While no one would be compelled to enter a hospital, such entry in a certified case should be a condition precedent to the granting of State aid to the dependents.

Dr. J. B. Cook maintained that the treatment of the sick poor as now enforced by the Ministry of Health is not in the parlous condition credited to it from some quarters. The infirmaries, of course, take in chronic cases, but they also take in numbers of patients for whom the voluntary hospitals have inadequate accommodation. The infirmaries have been referred to as "the rubbish-heaps of practice"; but Dr. Cook is glad to have such rubbish on his heap. The infirmaries, however, must be more adequately staffed with resident medical officers. Specialists should be available at each infirmary to act in consultation with the resident staff as required, and also to undertake special duties.

Dr. R. H. Cole praised the Poor-law infirmaries for the good work they have done in the treatment of mental disease, but admitted there is room for improvement.

According to Mr. Z. Cope, there is something to be said for separating the executive side from the side of medical and surgical treatment, and he believes also that the Poor-law infirmary offers a great opportunity for the development of post-graduate teaching.

Dr. A. Blackhall-Morison discussed medical politics somewhat generally, and protested strongly against the nationalisation of medicine. In his view the function of the Poor-law infirmary, however efficient it might be rendered by further officering, must be, in the future as in the past, to attend the true poor as distinct from the new rich. Those of the public who are not in this category of the poor can find all the medical attention necessary in their own domiciles, and he considers that only those who have drawn their conclusions from institutional, as distinguished from domiciliary, experience could think otherwise. These institutions should not be alienated from the purpose of their existence—namely, the care of the necessitous poor.

Dr. Nathan Raw, M.P., had no doubt that Dr. Addison's measure, so roughly handled in "another place," would be reintroduced, and as for the Poor-law infirmary, he held that the pauper class is disappearing. Representatives of trade unions declare that the poorer classes will accept neither charity from the voluntary hospitals nor relief from the Poor Law. The infirmaries which are no longer required for the destitute poor might be taken over by the health authority of the district and used as general hospitals, so as to be available for every member of the community. Legally, at present no one can enter a Poor-law infirmary save by order of the relieving officer or as a case of urgency. The whole procedure of taking the non-pauper classes into the Poor-law infirmary should be legalised by transferring the infirmaries to the municipal authorities by consent.

The President (Dr. Turtle), in summing up the discussion, concluded that if the linking-up of hospitals and Poor-law infirmaries can be legalised, that course seems to promise the best results, and each voluntary hospital might well be linked up with two infirmaries.

